# HSF4 regulates lens fiber cell differentiation by activating p53 and its downstream regulators

**DOI:** 10.1038/cddis.2017.478

**Published:** 2017-10-05

**Authors:** Meng Gao, Yuwen Huang, Ling Wang, Mi Huang, Fei Liu, Shengjie Liao, Shanshan Yu, Zhaojing Lu, Shanshan Han, Xuebin Hu, Zhen Qu, Xiliang Liu, Tinsae Assefa Yimer, Lifang Yang, Zhaohui Tang, David Wan-Cheng Li, Mugen Liu

**Affiliations:** 1 Key Laboratory of Molecular Biophysics of Ministry of Education, College of Life Science and Technology, Center for Human Genome Research, Huazhong University of Science and Technology, Wuhan, Hubei 430074, China; 2 State Key Laboratory of Ophthalmology and Visual Sciences, Zhongshan Ophthalmic Center, Sun Yat-sen University, Guangzhou 510060, China; 3 Key Laboratory of Kidney Disease Pathogenesis and Intervention of Hubei Province, Key Discipline of Pharmacy of Hubei Department of Education, Medical College, Hubei Polytechnic University, Huangshi, Hubei 435003, China

## Abstract

Cataract refers to opacities of the lens that impede the passage of light. Mutations in heat shock transcription factor 4 (*HSF4*) have been associated with cataract; however, the mechanisms regarding how mutations in *HSF4* cause cataract are still obscure. In this study, we generated an *hsf4* knockout zebrafish model using TALEN technology. The mutant zebrafish developed an early-onset cataract with multiple developmental defects in lens. The epithelial cells of the lens were overproliferated, resulting in the overabundance of lens fiber cells in hsf4^null^ zebrafish lens. Consequently, the arrangement of the lens fiber cells became more disordered and irregular with age. More importantly, the terminal differentiation of the lens fiber cell was interrupted as the organelles cannot be cleaved in due time. In the cultured human lens epithelial cells, HSF4 could stabilize and retain p53 in the nucleus to activate its target genes such as fas cell surface death receptor (*Fas*) and Bcl-2-associated X apoptosis regulator (*Bax*). In the hsf4^null^ fish, both p53 and activated-caspase3 were significantly decreased. Combined with the finding that the denucleation defect could be partially rescued through microinjection of p53, fas and bax mRNA into the mutant embryos, we directly proved that HSF4 promotes lens fiber cell differentiation by activating p53 and its downstream regulators. The data we presented suggest that apoptosis-related genes are involved in the lens fiber cell differentiation. Our finding that HSF4 functions in the upstream to activate these genes highlighted the new regulatory modes of HSF4 in the terminal differentiation of lens fiber cell.

Cataract is a major cause of adult blindness and congenital cataract is a major cause of childhood blindness.^1^ At least 44 genetic loci and over 40 genes have been linked to congenital or early-onset cataract.^2, 3, 4^ Heat shock transcription factor 4 (HSF4) has been associated with isolated cataract^5^ and it belongs to the heat shock transcription factor family (including HSF1, HSF2, HSF3 and HSF4). These HSFs can respond to various stress stimuli and protect cells against proteotoxic damage.^6, 7^ More importantly, HSFs are also involved in regulating differentiation and development.^8^ HSF4 differs from other HSFs in that it lacks C-terminal HR-C domain, which is responsible for the negative regulation of trimerization.^7, 9^ Moreover, whereas HSF1 and HSF2 are expressed in most tissues, HSF4 is predominantly expressed in the lens.^10, 11^

To investigate the function of HSF4, three *Hsf4* knockout mouse models have been constructed.^12, 13, 14^ All these three models developed early postpartum cataract. Histological analysis revealed that the nucleus and some cellular organelles of the secondary fiber cell persisted and the number of the epithelial cell increased. Associated with the altered biological processes, expression levels of the *Hsf4* target genes were significantly changed. These genes include the ones encoding heat shock proteins (Hsp70, Hsp60 and Hsp27), diverse types of *γ*-crystallins, various forms of fibroblast growth factors or the cognate receptor (FGF1, 2, 4, 7 and FGFR1), the beaded filament proteins (Bfsp1/2)^12, 13, 14^ and several other target genes (SKAP2, Vimentin).^15, 16^ Besides the knockout models, the HSF4 p.Arg116His mutation and the *Hsf4*(exon1)-DBD-EGFP hybrid gene transgenic mouse models have also been constructed. Both models developed postnatal lamellar cataract, which is similar to the phenotype caused by the mutations in the DBD of human *HSF4* gene.^17, 18^ These transgenic mice provide excellent models to study the lamellar cataract. Together, these studies revealed important functional aspects of the *HSF4* gene. However, the exact mechanisms by which *Hsf4* regulates lens development and whose mutations cause cataract still remain largely unknown.

Lens differentiation is a process through which an epithelial cell containing a full spectrum of cellular organelles is converted into a fiber cell characterized by the accumulation of high concentrations of lens-specific proteins and the loss of essential organelles.^19^ Recent studies demonstrated that lens differentiation is regulated by the same set of regulators responsible for the control of apoptosis.^19, 20^ These regulators include the tumor suppressor p53,^21, 22, 23, 24, 25, 26, 27, 28^ Bcl-2 family members,^25, 29, 30, 31, 32, 33^ caspase family members,^19, 34, 35, 35, 37, 38, 38, 40^ small heat shock proteins^32, 41, 42, 43, 44, 45^ and tumor necrosis factors.^46^ Tumor suppressor p53 has been implicated in regulating lens development. During mouse lens development, the expression of p53/Mdm2 was spatiotemporally regulated.^24^ Loss of p53 activity through expression of viral genes or the endogenous gene knockout induces posterior subcapsular cataracts.^21, 47, 48^ Besides, overexpressing human p53 in mouse lens led to microphthalmia.^22^ At the molecular level, p53 has been shown to regulate both major lens transcription factors c-Maf, Prox-1^26^ and differentiation-related crystalline genes.^49, 50^ In addition, p53 regulates numerous apoptotic genes, some of which are implicated in regulating lens differentiation. For example, Fas and Bax mediate both extrinsic and intrinsic death pathways, which are merged to activate the downstream executional caspase3. It has been discovered that caspase3 is a key regulator of lens development.^29, 30, 31, 32, 33, 34, 36, 38, 39, 51^ The *caspase3*^*−/−*^ mice developed cataract at the anterior lens pole.^51^ These findings indicated that p53 can regulate different sets of genes to control proliferation, apoptosis and differentiation of lens epithelial cells.

Our previous research discovered that HSF4 stabilizes p53 by inhibiting its ubiquitination and degradation. Through stabilizing p53, HSF4 can promote cell cycle arrest at the G1/S phase, thus protecting cells from overproliferation.^52^ In this study, we generated an *hsf4* knockout zebrafish line. The *hsf4* knockout zebrafish developed early-onset cataract with multiple cataractogenic defects, which were caused by uncontrolled cell proliferation and differentiation. More importantly, we demonstrate here that HSF4-oriented p53 is necessary and essential in regulating these activities. In absence of HFS4, p53 activity was downregulated and the expression of its downstream genes including Fas and Bax was significantly attenuated. As a result, both external and intrinsic apoptotic pathways were attenuated; thus, the conveyed caspase3 activity was significantly decreased, leading to incomplete organelle degradation. Thus, our results illustrate a fundamental mechanism regarding how HSF4 controls normal lens development and prevents cataractogenesis.

## Results

### Establishment of the *hsf4* knockout zebrafish using TALEN technology

**Figure 1:**
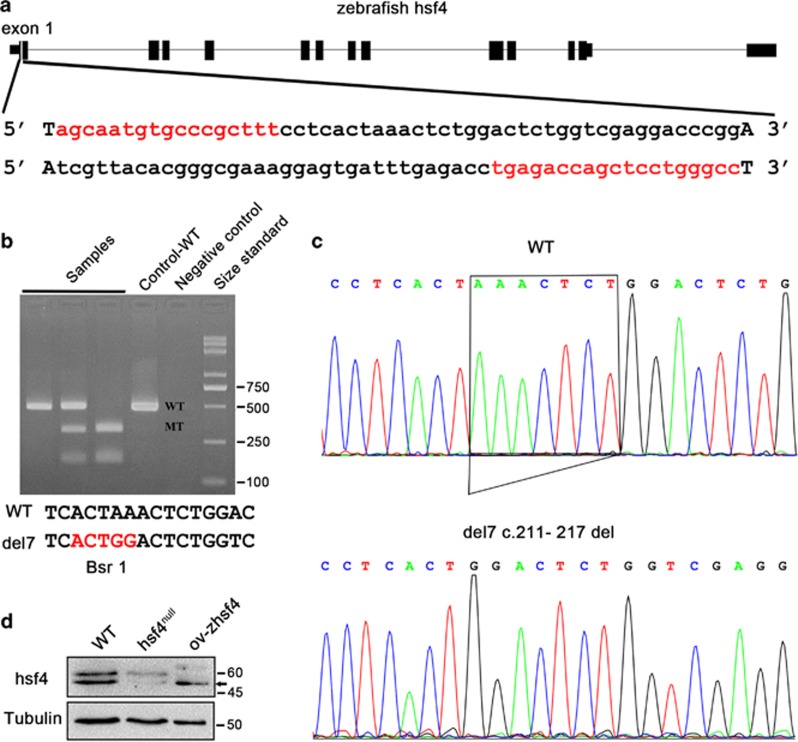
Establishment of the hsf4 knockout zebrafish. (**a**) All 13 exons of zebrafish *hsf4* and the TALEN target site were shown. The TALE sequences were highlighted in red. (**b**) Bsr1 assay. The upper picture was the genotyping results. PCR products from F2 zebrafish and wild type were subjected to the Bsr1 digestion. The wild-type allele cannot be digested, thus, only had one 466-bp band. The del7 allele could be digested by Bsr1, producing two bands that were 305 and 161 bp, respectively. Lanes (from left to right): three samples, WT control, negative control and size standard. The lower sequence showed the new Bsr1 restriction site generated by the 7-bp deletion in the mutant fish. (**c**) Sanger sequencing of WT and *hsf4* mutant fish (del7, c.211-217del). The 7-bp deletion was pointed out with a box. (**d**) Western blot analysis of the hsf4 protein in the WT and hsf4^null^ zebrafish. The zebrafish hsf4 protein expressed in eukaryotic cells was used as a positive control. Tubulin was a loading control and the ov-hsf4 sample from the HLECs was the positive control. The anti-hsf4 antibody could recognize the translated product of the transcript XM_009293553(424aa). The black arrow pointed to the hsf4 band

A pair of TALENs targeting exon1 of *hsf4* (XM_009293553) was designed on the website https://tale-nt.cac.cornell.edu/ to knockout *hsf4*.^53^ The selected target sites located in the DNA-binding domain are highly conserved between human and zebrafish^54^ (Figure 1a). Then, we used Fast TALE Assembly kit to construct hsf4-TALENs. TALEN mRNAs transcribed *in vitro* were microinjected into zebrafish embryos at the one- to two-cell stage. Positive embryos validated by sequencing were raised to adult and named F0 zebrafish. Their offsprings (F1) were screened by T7e1 enzyme and were sequenced to confirm the mutations. We identified a truncation mutation (c.211_217del, p.Lys24Glyfs10), named del7, which formed a new Bsr1 restriction site (Figure 1b). Subsequently, we crossed F1 to obtain homozygotes (F2). Genotypes of F2 were validated by Bsr1 cleavage (Figure 1b) and sequencing (Figure 1c). And then, western blot detection was performed to ensure that our *hsf4* knockout was effective. The result confirmed that no hsf4 protein existed in del7-mutant homozygotes (Figure 1d). Thus, the homozygous *hsf4* mutant zebrafish we acquired in this study are authentic hsf4^null^ zebrafish.

### *hsf4* knockout in zebrafish causes early-onset cataract

**Figure 2:**
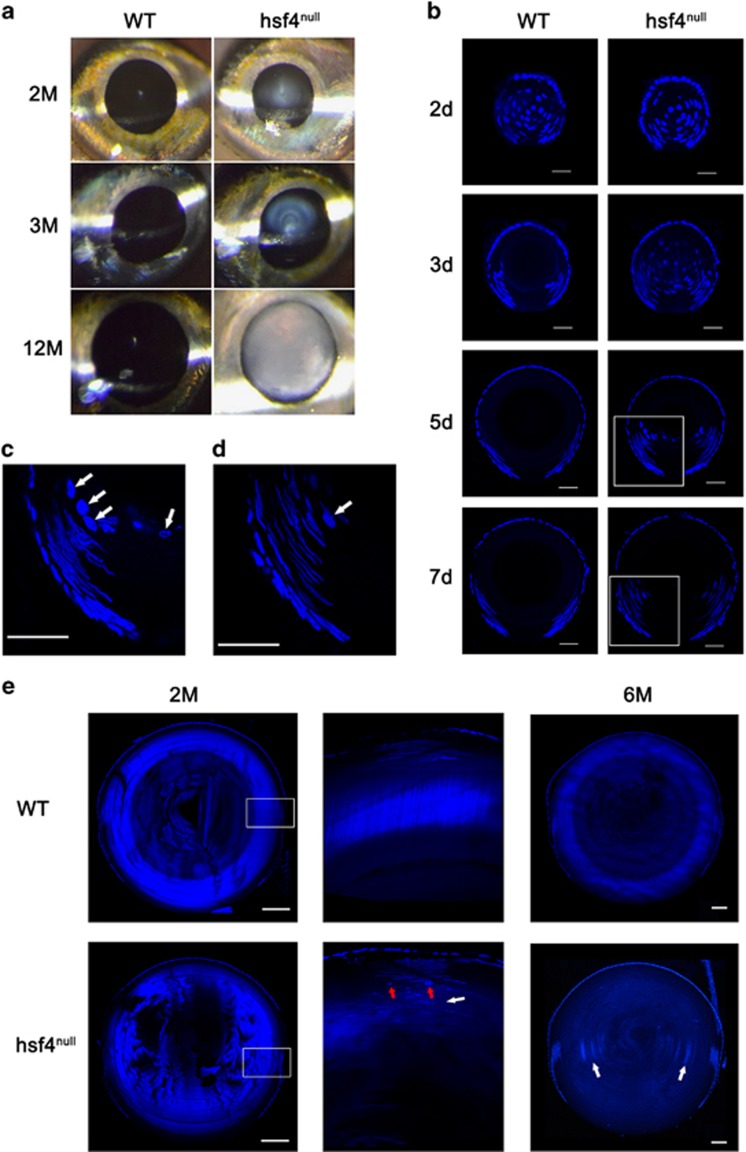
Knockout of *hsf4* in zebrafish caused early-onset cataract. (**a**) Slit lamp examination of the WT and hsf4^null^ zebrafish. Opacity of the eye lens could be detected from hsf4^null^ zebrafish at all the ages we examined. However, the WT lens were transparent. (**b**) DAPI staining of the WT and hsf4^null^ zebrafish lens at 2, 3, 5 and 7d. Scale bar: 20 *μ*m. (**c**) Enlarged image of white box in the lens of 5-d-old hsf4^null^ lens in **b**. (**d**) Enlarged image of white box in the lens of 7-d-old hsf4^null^ lens in **b**. The white arrows in **c** and **d** indicate the spherical nucleus. Scale bar: 20 *μ*m. (**e**) DAPI staining of the adult WT and hsf4^null^ zebrafish lens at 2 and 6M. The middle column is the enlarged images of white boxes in 2M WT and hsf4^null^ lens. The white arrows indicate the undegraded nucleus deposited in the hsf4^null^ lens. The red arrows indicate the spherical nucleus existing in hsf4^null^ lens. Scale bar: 500 *μ*m

A slit lamp examination of the hsf4^null^ zebrafish eye revealed clear presence of cataract formation at different stages including 2M, 3M and 12M (Figure 2a). By 12M, the hsf4^null^ zebrafish lens became completely opaque (Figure 2a). In contrast, the WT lenses were transparent at all ages examined (Figure 2a). Defects in degenerating organelles are major causes of cataract. Thus, by observing denucleation status we can detect the onset of this defect. Using semiquantitative reverse transcription and polymerase chain reaction, we first determined that hsf4 was highly expressed in eyes at 48 hpf (Supplementary Figure 1). Shortly after that, denucleation began at 50 hpf, indicating that hsf4 was critically important in this process. Consistent with the expression pattern, no significant difference was observed between WT and hsf4^null^ zebrafish before 48 hpf. After 2 dpf, great differences have been discovered. The WT zebrafish completed denucleation of the primary fiber cells before 3 dpf, and the secondary fiber cells successfully disintegrated their nuclei. In contrast, in hsf4^null^ zebrafish lens, almost all the primary fiber cells in the lens core contained nuclei at 3 dpf. By 5 dpf, there were some nuclei still remaining in the lens core. The denucleation of the primary fiber cells eventually completed at 7 dpf, which indicated that the denucleation of the primary fiber cells was delayed but not abrogated when *hsf4* was deleted (Figure 2b).

The denucleation of the secondary fiber cells seemed more severely affected within hsf4^null^ zebrafish. By the ages of 2 and 6M, we discovered that most of the fiber cells in hsf4^null^ lens contained intact nuclei, which were deposited together and even reached the most inner part of the lens. In the WT lens, however, the denucleation of the fiber cells was normal for we could only detect nuclei in the superficial differentiating fiber cells (Figure 2e). To our surprise, we found some spherical nuclei in the secondary fiber cells (Figures 2c–e). When the lens fiber cell differentiation initiates, nuclei start to elongate and become ovoid. Studies in bovine and chicken revealed that the volume and shape of the nuclei changed during disintegration.^55, 56^ Normally, the breakdown of the nucleus is a very rapid process; thus, the degenerating spherical nuclei are seldom found. The spherical nuclei we detected in the hsf4^null^ lens maybe the fiber cells that were about to degrade. As the hsf4^null^ lens contained an overwhelming majority of secondary fiber cells with ovoid nuclei, we reasoned that only a small proportion of the secondary fiber cells could disintegrate their nuclei in an hsf4-independent manner. Without hsf4, the denucleation of the secondary fiber cells appeared to come to a standstill.

### hsf4 loss interrupts terminal differentiation of Zebrafish lens fiber cells

**Figure 3:**
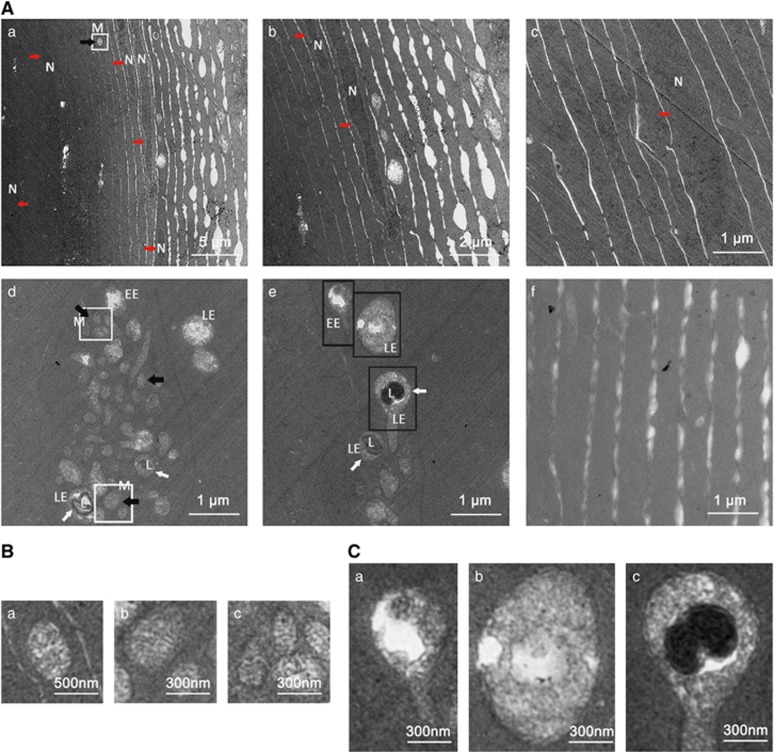
Interrupted organelle degradation in the lens fiber cells of hsf4^null^ zebrafish. (**A**) Transmission electron micrographs of lens fiber cells from 5-M-old hsf4^null^ zebrafish and WT zebrafish. Nucleus (red arrow), mitochondria (black arrow) and vesicular structures (white arrow) are obvious features in hsf4^null^ lens fiber cells (a–e). In addition, the abbreviations including N (nucleus), M (mitochondria), EE (early endosome), LE (late endosome) and L (lysosome) were marked in the picture to emphasize the organelles' persistence. The connection between the fiber cells is loose in the cortical fiber region (a and b). (c) The fiber cells in the inner region of lens have an intact nucleus. (d and e) The mitochondria and vesicular structures including EE, matured LE and LE/lysosome fusion structure depositing in the lens fiber cells. The white arrows indicate the LE/lysosome fusion structure. (f) Showing that no cell apparatus existed in the WT lens fiber cells. (**B**) Enlargement pictures of the white box labeling areas showing that intact mitochondria existed in lens fiber cells. Subpanel a corresponds to white box in **A**a. (b and c) Upper and lower white boxes in **A**d, respectively. (**C**) Enlargement pictures of black box labeling areas in **A**e showing the endosomes at different stages. (a) EE, (b) LE and (c) LE/lysosome fusion structure

Previous research discovered that nuclei were preserved in the differentiating fiber cells as confirmed in hsf4^null^ zebrafish; it is not clear whether the cellular non-nucleus organelles are also preserved in *hsf4* knockouts. Thus, we performed transmission electron microscopy of 5-M-old hsf4^null^ and WT zebrafish. As shown in Figure 3Af, the WT lens fiber cells were transparent and organelle-free, i.e., no dark stained structures interfering with the focusing of inside structure. In contrast, the fiber cells of the hsf4^null^ zebrafish contained intact organelles, mitochondria, endosome and lysosome besides nuclei, suggesting that organelle degradation was also interrupted without hsf4 (Figure 3Aa–e). In the inner part of the lens, we could also detect intact nucleus (Figure 3Ac) and also observed that the connection between cortical lens fiber cells was loose (Figure 3Aa and b. In addition, the mutant fiber cells contained clustered vesicular structures (Figure 3Ad and e). These structures were early endosome, late endosome (LE) and LE–lysosome fusion body as observed from enlarged figures (Figure 3c). Usually, LE will be degraded when it is translocated to and fused with lysosome at the perinuclear region. In the hsf4^null^ zebrafish lens, however, the deposited endosomes were mislocalized and left undegraded. Together, our study clearly revealed that the non-nucleus organelle degradation was also interrupted in the hsf4^null^ lens fiber cells.

### hsf4 loss interrupts balanced cell proliferation and differentiation, which leads to pathological lens fiber architecture in Zebrafish lens

To testify whether the number of the lens epithelium cell was also increased in hsf4^null^ zebrafish, we performed paraffin section and DAPI staining on 5-M-old zebrafish. Under microscopy observation, we confirmed that the epithelial cells were overproliferated. In adult lens, the proliferation activity at the central region of the lens epithelium is extremely low; thus, overproliferation was more obvious and significant in the germinal zone (Supplementary Figure 2).

**Figure 4:**
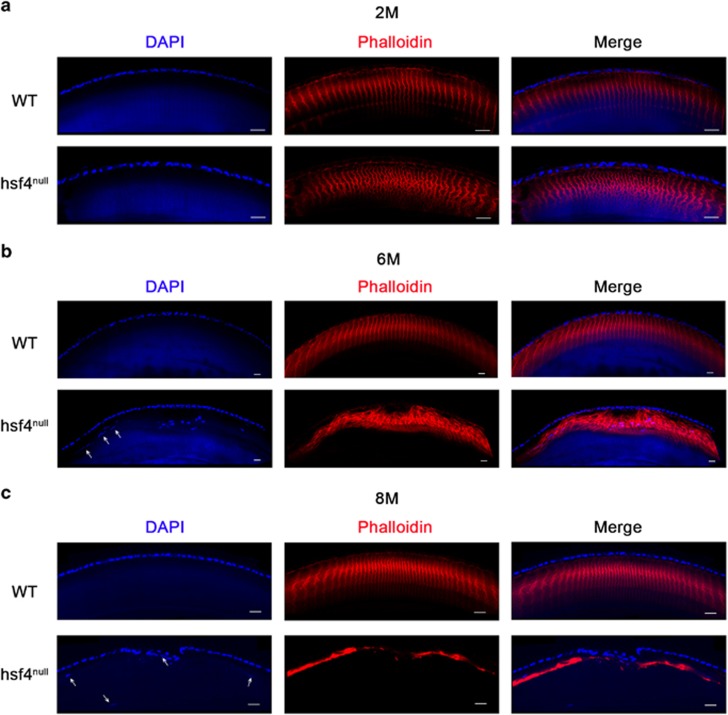
Progressively disordered arrangement of lens fiber cells in the in the hsf4^null^ zebrafish lens. Immunostaining the WT and hsf4^null^ zebrafish lens from different ages with antiphalloidin antibody. (**a**) The signals are more denser in the hsf4^null^ zebrafish than the WT zebrafish at 2M of age. (**b**) The fiber cell structures becoming more disorganized in the *hsf4*^*null*^ zebrafish at 6M of age. (**c**) The irregular signal disappeared in the hsf4^null^ zebrafish at 8M of age. The white arrows indicate the mislocated fiber cell nucleus in the anterior region of the lens. Scale bar: 20 *μ*m

We performed frozen section and phalloidin staining to determine whether the overproliferated cells at the germinal zone would produce excessive number of lens fiber cells. Under microscopy observation, we found that the fiber cell was labeled with phalloidin immunofluorescence, forming a hexagonal fluorescence circle. However, these circles stack together to generate a regular structure (Supplementary Figure 4). The WT lenses from every time point we checked displayed the regular arrangement pattern. In contrast, notable differences showed in hsf4^null^ lenses. Compared with the WT lens, the fluorescence signals in hsf4^null^ lens were denser but well organized at 2M, suggesting that more fiber cells existed in hsf4^null^ lens (Figure 4a). At 6M, the standard arrangement pattern was severely interrupted (Figure 4b). By the age of 8M, the signals almost completely disappeared (Figure 4c). In addition, we could also find some nuclei mislocalized in the anterior region of the 6 and 8M hsf4^null^ lenses. The above findings indicated that the arrangement of the fiber cells was disturbed (Figures 4b and c). Together, these results suggest that the overproliferation of the lens epithelial cells contributes to the excessive accumulation of fiber cells in the hsf4^null^ lens with age, which causes chaotic arrangement of the fiber cells in the limited space, leading to cataractogenesis.

### HSF4-governed p53 stability and nuclear localization are essential to activate the *Fas*-mediated apoptotic signaling pathway

**Figure 5:**
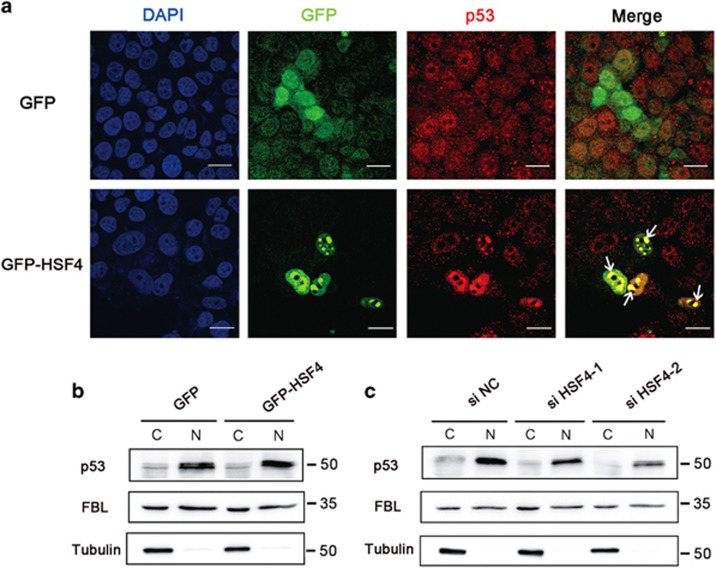
HSF4 stabilizes and orients p53 in the nucleus to initiate. (**a**) p53 is retained in the nucleus by HSF4. HLECs were transfected with GFP-tagged HSF4 (green) plasmids and negative control GFP vectors, respectively. Cells were harvested 48 h after transduction followed by immunofluorescence stains with anti-p53 antibody. Arrows indicated the overlap of the p53 signals (red) and the HSF4 signals (green). Scale bar: 10 *μ*m. (**b**) The nuclear distribution of p53 was increased when HSF4 was overexpressed in HLECs. (**c**) Nuclear-oriented p53 was decreased when HSF4 was silenced in HLE cells. The cytoplasmic and nuclear proteins were separated and subjected to western blot detection. Tubulin and FBL were used as specific markers for the cytoplasm and nuclear components, respectively

Continued with our previous research, we further investigated the roles of HSF4 and p53 in fiber cell differentiation. Firstly, we confirmed that HSF4 could stabilize p53 in human lens epithelial cell lines (HLECs). As expected, cells expressing GFP-HSF4 showed a stark enrichment of endogenous p53 in the nuclei (Figure 5a). Moreover, the endogenous p53 was predominantly localized in nuclei in a diffused pattern in cells transfected with GFP vector. In contrast, p53 was more focused and oriented in the nuclei but not in the cytoplasm of the cells expressing GFP-HSF4 fusion protein (Figure 5a). Western blot analysis of p53 levels in the separated nuclear and cytoplasmic fractions confirmed our immunofluorescence data (Figure 5b). On the other hand, p53 was obviously decreased in the nucleus when HSF4 was silenced using either of two HSF4-specific siRNAs (Figure 5c).

**Figure 6:**
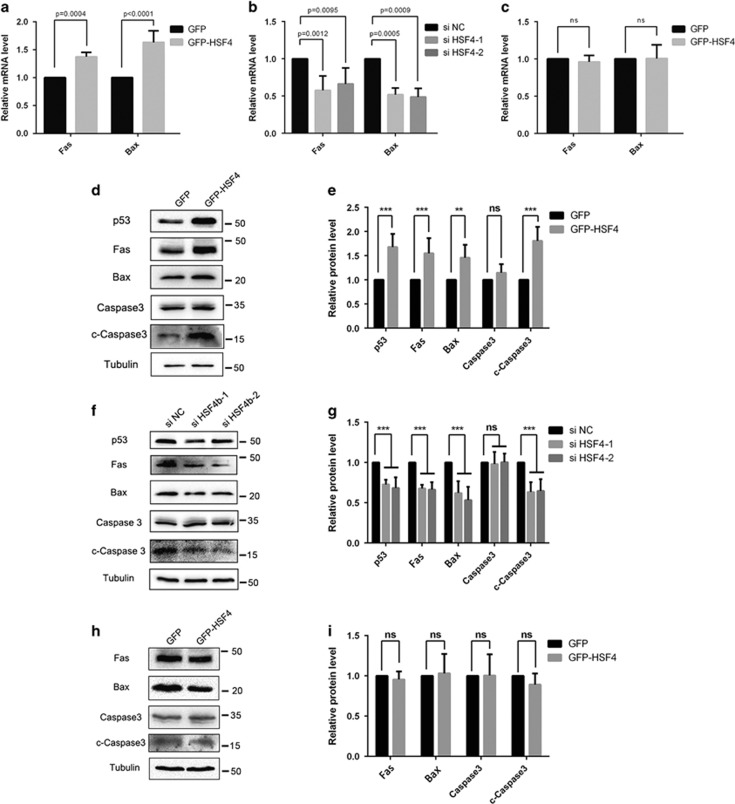
HSF4 can initiate the Fas-mediated apoptosis signaling pathway in HLECs. (**a**) Fas and Bax were upregulated at the mRNA level when HSF4 was overexpressed in HLECs. GFP-tagged HSF4 and negative control GFP plasmids were transfected into HLE cells. And then cells were harvested for RNA extraction and real-time PCR detection. Five independent experiments were performed and the relative mRNA levels were normalized to *β*-actin. (**b**) Fas and Bas were downregulated at the mRNA level when HSF4 was silenced in HLECs. The HSF4-specific siRNAs were transfected into HLECs. And then cells were harvested for RNA extraction and real-time PCR detection. Five independent experiments were performed and the relative mRNA levels were normalized to *β*-actin. (**c**) The upregulation of FAS and BAX was p53-dependent. GFP-tagged HSF4 and negative control GFP plasmids were transfected into H1299 cells. And then cells were harvested for RNA extraction and real-time PCR detection. Five independent experiments were performed and the relative mRNA levels were normalized to *β*-actin. (**d**) HLECs were transfected with GFP-HSF4 plasmids and negative control GFP vectors. Cells were harvested 48 h after transduction followed by protein extraction and western blot detection. (**e**) Statistical analysis of western blot detection result in **d**. The relative protein level was normalized by *α*-tubulin. Three independent experiments were performed. (**f**) The HSF4-specific siRNAs were transfected into HLECs. And then cells were harvested for protein extraction and western blot detection. (**g**) Statistical analysis of western blot detection result in **f**. The relative protein level was normalized by *α*-tubulin. Three independent experiments were performed. (**h**) H1299 cells were transfected with GFP-HSF4 and negative control GFP vectors. After 48 h later, cells were harvested for protein extraction and western blot detection. (**i**) Statistical analysis of western blot detection result in **f**. The relative protein level was normalized by the *α*-tubulin. Three independent experiments were performed. The overexpression and RNA-interfering efficiency were validated simultaneously, and the results were listed in Supplementary Figure 5 and Supplementary Figure 6

As previous studies have revealed that apoptotic regulators are implicated in regulation of lens differentiation, we tested *Fas* and *Bax*, two target genes of p53 that encode proteins mediating extrinsic and intrinsic apoptotic pathways. First, through real-time PCR analysis, we found out that both Fas and Bax were significantly upregulated when HSF4 was overexpressed in HLECs (Figure 6a). In contrast, if HSF4 was silenced both Fas and Bax mRNA were significantly decreased (Figure 6b). Western blot results confirmed the changes of Fas and Bax at the protein level parallel with them at the mRNA level (Figures 6d–g). In addition, we noticed that the executor caspase3 was significantly activated in the cells expressing GFP-HSF4 compared with GFP control (Figure 6d). Consistent with this observation, the cleaved-caspase3 was decreased when we silenced HSF4 in HLECs (Figure 5f). Second, we determined whether HSF4-mediated upregulation of Fas and Bax was p53-dependent. To do so, the p53-null H1299 cells were transiently transfected with GFP vector or GFP-HSF4 fusion protein expression constructs. After 48 h, these cells were harvested for real-time PCR and western blot analysis. The results showed that both Fas and Bax were not altered at either mRNA or protein level (Figures 6c and h). Increased activation of caspase3 could not be detected in H1299 cells expressing GFP-HSF4 (Figure 6h). Thus, our results demonstrated that the activation of the Fas-mediated apoptotic signaling pathway by HSF4 was p53-dependent.

### hsf4 loss can be partially rescued by overexpression of hsf4, p53 and fas

**Figure 7:**
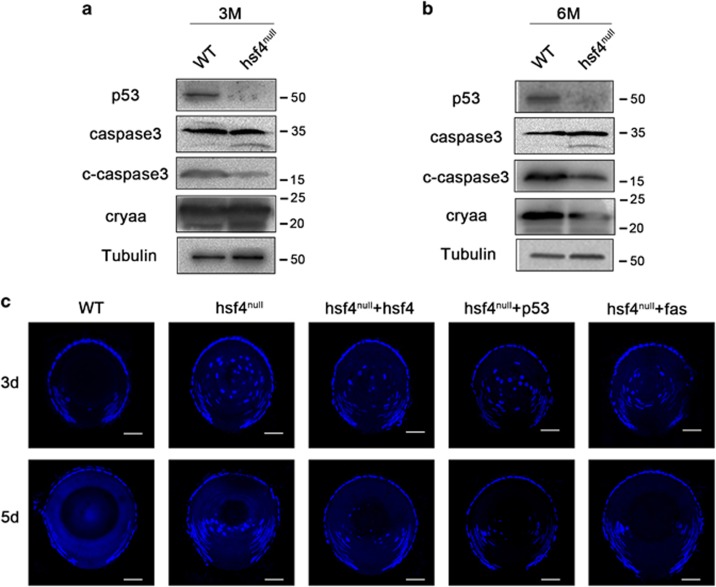
hsf4 loss can be partially rescued by overexpression of hsf4, p53 and fas. (**a**) Three-M-old WT and hsf4^null^ zebrafish lenses were isolated and subjected to protein extraction. Western blot results indicated that p53 and the activation of caspase3 was significantly decreased in the hsf4null zebrafish. (**b**) Western blot analysis of the proteins from 6-M-old WT and hsf4^null^ zebrafish lenses. The results revealed that p53 and cleaved-caspase3 were also obviously decreased in the 6-M-old hsf4^null^ zebrafish lens. (**c**) Overexpression of hsf4, p53 and fas could partially rescue the denucleation defect in hsf4null zebrafish lens. The *hsf4*, *p53* and *fas* mRNA was microinjected into the hsf4^null^ zebrafish embryos. The denucleation status was checked at 3 and 5d, respectively, through the DAPI staining. Obviously, decreased numbers of nuclei were presented in the lens injected with *hsf4*, *p53* and *fas* mRNA at both 3 and 5d. Scale bar, 20 *μ*m

As HSF4 can regulate p53 stability and control Fas and Bax in a p53-dependent manner, we next sought to determine the functions of HSF4 regulation of p53 and its downstream target genes in governing lens development. To do so, we extracted proteins from hsf4^null^ and WT zebrafish lenses. Western blot analysis revealed that p53 was significantly downregulated in hsf4^null^ lens (Figures 7a and b). As p53 was downregulated, the activation of caspase3 was also significantly decreased, whereas the protein level of procaspase3 was not affected at two different time points we tested (Figures 7a and b). Together, these results demonstrated that HSF4 can induce caspase activation by stabilizing p53 *in vivo*.

To further test the functions of HSF4-controlled p53 and its target gene in regulating lens differentiation, we overexpressed these genes to determine whether they can rescue the denucleation defect in hsf4^null^ lens. To do so, we collected hsf4^null^ embryos and performed microinjection using hsf4, p53 and fas mRNA. As we have shown that the hsf4^null^ zebrafish displayed obvious defects in degrading the nuclei of the lens fiber cells after 3 dpf, we collected the injected embryos at 3 and 5d. Through frozen section and DAPI staining, we found that hsf4, p53 and fas could partially rescue the denucleation defect in hsf4^null^ lens (Figure 7c). These results demonstrated that hsf4 can regulate lens fiber cell differentiation by regulating p53 and its downstream apoptotic regulators.

## Discussion

By constructing and analyzing the hsf4^null^ zebrafish, we demonstrated the following: (1) hsf4^null^ zebrafish develops an early-onset cataract and the pathologic condition remained with age; (2) the overproliferation of the lens epithelial cells contributes to excessive accumulation of the lens fiber cells, which interrupt lens normal arrangement pattern and lead to cataractogenesis; (3) at the cellular level, the denucleation of the primary fiber cell is much delayed in hsf4^null^ lens, and this becomes even more severe in the differentiation progress of secondary fiber cells. Both nuclei and other cellular organelles including mitochondria, endosome and lysosome cannot be disintegrated. This is another reason for cataractogenesis; (4) at the molecular level, by stabilizing p53, HSF4 can regulate both extrinsic and intrinsic apoptotic pathways to mediate its control of lens differentiation. Loss of HSF4 leads to downregulation of p53, inactivation of the p53-dependent death with clear attenuation of caspase3 activation and eventual halt of the lens differentiation. Together, our results reveal a fundamental mechanism by which HSF4 controls normal lens development and prevents cataractogenesis.

### HSF4 is a major factor governing lens differentiation

The hsf4^null^ zebrafish developed an early-onset cataract with multiple defects as we summarized above. However, while the studies from the laboratories of both Nakai^12^ and Mivechi^13^ reported clear microphthalmia, we did not notice any obvious changes in eye size. Our results are consistent with the Hsf4^null^ mouse model reported by Hu^14^ and the transgenic mouse models.^17, 18^ We noticed that the two models with microphthalmia were generated from C57Bl/6 mice, whereas the mouse models with normal eye size were generated from S129 mice. We speculate that the difference of the genetic background causes inconsistency in the observed eye size. Of course, other possibilities could not be ruled out.

It is noteworthy that besides the nuclei and mitochondria, we found other membrane-bound organelles, including endosomes and lysosomes, persisting in hsf4^null^ lens. Under normal conditions, the matured LEs translocate to and fuse with lysosome at perinucleus regions to proceed to the unidirectional degradative pathway. Without hsf4, the LEs and lysosomes were mislocated. When the distribution of endosome and lysosome disperses throughout the cytoplasm, the degradation activity will be affected. Our previous studies have shown that HSF4 can upregulate lysosome activity in HLECs.^57^ Furthermore, the p53 and Bax could increase the lysosome membrane permeabilization to trigger cell death. Thus, the accumulation of matured LE in the hsf4^null^ lens might be caused by the decreased activity of lysosome. However, the exact mechanisms of why the distribution of the endosomes becomes dispersal and how hsf4 affects this process remain to be further investigated.

### HSF4 stabilizes its key function mediator p53 to regulate lens differentiation at different levels

The balanced p53 activity in the ocular lens is essential for normal lens development. In ocular lens, HSF4 is one of the key factors to stabilize p53. It does so in different ways. First, HSF4 can stabilize p53 through the direct interaction with each other.^52^ Secondly, HSF4 relies on its target gene products *α*B-crystallin and vimentin to stabilize p53.^16^ *α*B-crystallin can interact with p53 to stabilize the later.^58^ The HSF4-regulated upregulation of p53 can initiate cell apoptosis signaling, leading to the apoptotic cleavage of vimentin. The cleavage of vimentin leads to the release of cytosolic p53 for nuclear translocation, thus stabilizing p53 and amplifying cell death signal.^59^ Finally, HSF4 can stabilize p53 through p53 target genes. The HSF4 target, *α*B-crystallin gene, is also regulated by p53 and *α*B-crystallin can interact with p53 to stabilize the later.^58^

### HSF4 regulates p53-mediated extrinsic and intrinsic death pathways to control lens differentiation

It has been well documented that the same set of regulators can be used for control of both apoptosis and lens differentiation.^19, 20^ In this regard, the HSF4-stabilized p53 has an important role. First, p53 can regulate major lens transcription factors such as C-Maf and Prox-1 to promote lens differentiation.^26^ Second, it directly regulates the differentiation-related genes including members of the *α*-, *β*- and *γ*-crystallin genes.^49, 50, 58^ The promoted expression of *β*- and *γ*-crystallin genes are symbols of lens differentiation, thus helping cell maturation in lens fiber. In addition, p53 can regulate a large of number apoptosis regulators. Among these regulators, Bcl-2, Bax and Bak are members of the intrinsic death pathways, which have been shown to participate in lens differentiation.^29, 30, 31, 32, 33^ Our study demonstrated that HSF4 can upregulate Bax, and participate in the p53-mediated apoptotic program during lens development. Moreover, our study provided evidence that Fas, another p53 target gene and member of the extrinsic death pathway, is also regulated by HSF4. Fas when overexpressed in LEC can trigger cell death and DNA fragmentation.^60^ Our data showed that injection of fas mRNA can rescue the denucleation defect caused by hsf4 deletion. Finally, the p53-mediated apoptotic signal pathways activate the executional caspase3, which was found upregulated and had a role during the development of lens in rat,^19, 34, 36^ mouse,^37, 38, 39^ chicken^30, 31, 33^ and zebrafish.^40^ Here, we demonstrated that knockout of *hsf4* significantly decreased the activation of caspase3. It has been discovered that, during erythrogenesis, the volume of the nucleus was condensed because of the formation of a caspase3-mediated nuclear opening.^61^ Erythrogenesis shares some similarities with lens fiber cell differentiation. The same phenomenon has been observed in the hsf4^null^ zebrafish lens, suggesting that the nuclear condensation in the lens fiber cells may also proceed in a similar way.

Besides its control of the above-mentioned pro-apoptotic regulators, HSF4 is also implicated in the regulation of anti-apoptosis pathway. Heat shock protein *α*B-crystallin is a target gene of HSF4 and an important regulator against apoptosis. In mouse lens, *α*B-crystallin co-localized with procaspase3 in the differentiating secondary fiber cells of the transition zone.^62^ Furthermore, the expression of Bax was elevated in the fiber cells, which were undergoing terminal differentiation.^33^ Moreover, the co-localization of Bax and *α*B-crystallin has also been discovered in these fiber cells. By interacting with *α*B-crystallin, the activation of procaspase3 was repressed and the translocation of Bax from the cytoplasm into the mitochondria was also inhibited.^62^ Together, these findings indicate that the regulators including p53, Fas, Bax and caspase3 can form a regulating system with some other target genes of HSF4. This regulating system organized by HSF4 is necessary for the fiber cell differentiation by maintaining a fine balance between pro-apoptosis and anti-apoptosis pathways.

The molecular mechanisms mediating organelle degradation still remain a puzzle and is the focus for the researchers in the field. Our work presented here support the idea that apoptosis-related genes including p53, Fas and Bax are involved in regulating lens fiber cell differentiation. More importantly, we discovered that HSF4 is responsible for activating the p53-mediated apoptosis signaling pathways in the terminal differentiation of lens fiber cell. Our work provided some new clues for revealing why HSF4 mutations can result in the failure in the lens fiber cell differentiation and cause cataract.

## Materials and methods

### Zebrafish maintenance and breeding

Our research was approved by the Ethics Committee of Huazhong University of Science and Technology. Zebrafish of AB line were housed in recirculating water system (pH 6.6–7.4, 26–28.5 °C) with a daily cycle of 14 h of light and 10 h of dark. We fed the adult zebrafish with fresh brine shrimps three times a day. For the baby fish, we also fed three times a day with live paramecia when they are after 5 dpf, paramecia mixed with brine shrimps after 10 days and only the brine shrimps after 30 dpf. The males and females (1 : 1 or 1 : 2) were separated in the crossing cages with a plastic divider in the early evening before the mating. They were kept undisturbed through the whole night and then we mixed them at about 0900 hours. After spawning, we collected the eggs and bathed them with embryo medium. The embryos were kept in an incubator (28.5 °C) for 72 h until the larvae were hatched. During this period, the medium was refreshed and the debris was removed every day.

### TALEN construction and mRNA synthesis

The gene sequence information for zebrafish *hsf4* (NC_007129) was acquired from NCBI (http://www.ncbi.nlm.nih.gov/). We used the online tools TAL Effector Nucleotide Targeter2.0 (https://tale-nt.cac.cornell.edu/) to design TALENs that target the exon1 of *hsf4*; the left target sequence was 5′-AGCAATGTGCCCGCTTT-3′ and the right was 5′-CCGGGTCCTCGACCAGAGT-3′. The plasmids of the TALENs were assembled by using the Fast TALE TALEN kit (Sidansai Biotechnology, Shanghai, China) according to the instructions provided by the Company.

### Microinjection and genotyping

A pair of TALEN RNAs was mixed and final concentration of each arm was 100ng/*μ*l. Then, the mixed mRNA was injected into one/two-cell-stage embryos. Three days after the injection, 10–15 embryos were collected for extracting genomic DNA. The target site of *hsf4* was amplified and the product was digested by T7E1 (ViewSolid Biotech, Beijing, China) to verify the mutation as described in the text.

### Cell culture, plasmids, transfection and small interfering RNA

The HLEC and the human lung carcinoma cell line (H1299) we used in this study were cultured in Dulbecco's modified Eagle's medium (DMEM, Gibco, Grand Island, NY, USA) containing 10% fetal bovine serum (FBS, Gibco). The plasmids we used to express human HSF4 and p53 have been illustrated in our published work.^53^ To construct the zebrafish *hsf4* eukaryotic expression vector, we extracted total RNA from the zebrafish lens. Through the reverse transcription, we obtained cDNA of the zebrafish lens. Using the lens cDNA as a template, we got the whole-lens zebrafish *hsf4*. After we got the zebrafish *hsf4*, we cloned it into the p3XFLAG-CMV-7.1 vector. Plasmids were transfected into HLECs and H1299 cells with Lipofectamine 3000 Reagent (Thermo Fisher, Wilmington, DE, USA) in Opti-MEMI Reduced SerumMedium (Gibco) and incubated for 6 h. After 48 h, cells were harvested for RNA and protein extraction. HSF4-specific interfering RNA was purchased from RiboBio, Guangzhou, China. The HSF4 siRNA duplexes were as follows: No1: 5′-GCAAGCUGAUCCAGUGUCU-3′ and No2: 5′-CGCCAACUCAACAUGUACG-3′. Stealth RNAi-negative control (NC) was used as a NC. The exact procedures for HSF4 silencing were performed as we have illustrated in our published paper; overexpression and knockdown of various genes were conducted as described before.^52^

### Real-time PCR

Total RNAs were extracted from the cultured cells using RNAiso Plus reagent (Takara, Kusatsu, Japan). The RNA (1 *μ*g) was reverse-transcribed into cDNA using MMLV reverse transcriptase (Invitrogen) andoligo(dT) primer (Takara). We took these cDNAs as templates to detect the mRNA levels of the specified genes by quantitative PCR, which were performed on the StepOnePlus real-time PCR System (Life Technologies, Carlsbad, CA, USA) using AceQ qPCR SYBR Green Master Mix (Vazyme, Nanjing, China). The data were analyzed using the 2ΔΔCT method in the StepOne software (version 2.3, ABI, Carlsbad, CA, USA). Significance was determined by two-tailed Student’s *t*-test.

Following primers have been used for the RT-PCT study:

HSF4: 5′-GAGATACCTGACAGGGGGCCTCTG-3′ and 5′-GGAACCAAGGGCTGCATCAAGGAC-3′,

p53: 5′-GGCCCATCCTCACCATCATCACA-3′ and 5′-TGCGCCGGTCTCTCCCAGGA-3′,

Fas: 5′-GGCTTTGTCTGTCTCCT-3′ and 5′-CCTACCTCTGGTTCTTACGT-3′,

Bax: 5′-GAAGTCCAATGTCCAGCCCA-3′ and 5′-AGTGTCTCAAGCGCATCGGG-3′,

Actin: 5′-TGACGTGGACATCCGCAAAG-3′ and 5′-CTGGAAGGTGGACAGCGAGG-3′.

### Frozen section

The eyeballs were isolated from the adult zebrafish and were fixed in the 4% paraformaldehyde (diluted in PBS) immediately. The eyes could be fixed at room temperature for 4–6 h or at 4 °C overnight. For the next step, we washed the tissue with PBS for three times and 15 min each time. The sucrose was diluted to a final concentration of 30% in the PBS. The zebrafish eyeballs were dehydrated in the 30% sucrose solution overnight at 4 °C. The tissue settled down at the bottom of the tubes when the dehydration was completed. And then, we embedded the eyeball in the OCT compound and then frozen them at −25 °C. Embedded tissues were sliced along the vertical meridian of each eyeball and sectioned each slice to 20 *μ*m thickness. For the small fish that was <10 days of age, the whole body was fixed for the frozen section and each slice was 8 *μ*m thick.

### Immunofluorescence and DAPI staining

We performed immunofluorescence on the cryosections and the cultured cells. For the cryosections, they were dried at room temperature to protect them from being detached. For the next step, the sections were washed with PBS for 10 min to remove the OCT compound, permeabilized in PDT (PBS containing 1% DMSO and 0.1% Triton X-100) for 10 min and blocked with 10% normal goat serum in PBDT (PDT containing 1% BSA) for 1 h at room temperature. The Alexa Fluor 568 phalloidin (Invitrogen) was diluted with PBS at the ratio of 1 : 50. The diluted phalloidin was added onto the slides and then incubated for 2 h at 37 °C. The slides were washed three times with PBS. DAPI was used to label the nucleus at the concentration of 5 *μ*g/ml. The sections were then rinsed three times with PBS, and mounted with a glycerol-based liquid mountant under coverslips. For the cultured cells, we washed the cells with PBS for three times to remove the medium and fixed them in 4% paraformaldehyde and permeabilized with 0.5% Triton X-100 for 15 min. The cells were blocked for 1 h at the room temperature with 5% BSA in PBS and then incubated with p53 primary antibody (1 : 100, Proteintech, Wuhan, China) overnight at 4 °C. Afterward we removed the primary antibody through washing them with PBS for three times. The cells were incubated with the secondary antibody conjugated with Alexa Fluorescence 568 (1 : 1000, Invitrogen, Carlsbad, CA, USA) in 37 °C for 1 h. Nuclei were stained with DAPI (1 : 5000, Sigma, St. Louis, MO, USA). Fluorescence images were acquired using a confocal laser-scanning microscope (FluoView FV1000 confocal microscope, Olympus Imaging, Tokyo, Japan).

### Transmission electron microscopy

Adult zebrafish eyes were isolated and fixed in 2.5% glutaraldehyde in 0.1 M PBS buffer (pH 7.0) overnight at 4 °C. After three washes (15 min each) with 0.1 M PBS buffer, the eyes were further fixed in 1% osmium tetroxide in 0.1 M PBS buffer for 2 h at room temperature. After three washes (15 min each) with 0.1 MPBS buffer, the eyes were dehydrated in 50, 70, 80, 90, 95 and 100% ethanol successively (20 min each) and incubated in acetone for 20 min at room temperature. The eyes were treated with 50% (1 h), 75% (3 h) and 100% (overnight) epoxy resin (mixed with acetone, v/v), and then heated at 70 °C overnight. Embedded eyes were sliced to ultrathin sections (70 nm) using an Reichert-Jung ultramicrotome (Leica, Wetzlar, Germany). Sections were stained with 3% uranyl acetate and 3% lead citrate for 15 min and were visualized with a transmission electron microscope system (HT7700, Hitachi, Tokyo, Japan).

### Western blot

For the cultured cells and the lens isolated from the eyeballs, both were lysed in RIPA cell lysis buffer (Beyotime, Shanghai, China) with cocktail protease inhibitor (Roche, Basel, Switzerland). After the ultrasonic decomposition and centrifugation, the protein extraction was completed. Protein concentration was determined using the BCA Protein Assay Kit (Beyotime). We mixed the protein lysates with loading buffer and boiled them for 10 min at 100 °C. And then, we placed the protein samples on the ice for 3 min before loading. The protein samples were separated by 12% SDS-PAGE and transferred to nitrocellulose membranes (Millipore, Danvers, MA, USA). Then, we blocked the membranes in the 5% skim milk dissolved in TBST buffer (20 mM Tris–HCl, 150 mM NaCl, 0.05% Tween 20, pH 7.6) for 1 h at the room temperature. For the next step, we washed the membranes with TBST buffer and incubated them with the diluted solution of primary antibodies overnight at 4 °C with gentle agitation. And then we washed the membranes with TBST buffer three times for 5 min each time. To visualize the result, we incubated the membranes in HRP-conjugated secondary antibodies (1 : 20 000; Thermo, Wilmington, DE, USA) for 2 h at room temperature. After incubation, the membranes were washed for another three times (5 min each time) with TBST buffer. The signals were detected using a ChemiDoc XRS (Bio-Rad Laboratories, Berkeley, CA, USA), with Super Signal Sensitivity Substrate (Thermo), and quantified with Quantity One software (Bio-Rad Laboratories, Berkeley, CA, USA).

### Statistical analysis

All experiments were repeated at least three times. Significance was determined by two-tailed Student’s *t*-test.

## Publisher’s Note:

Springer Nature remains neutral with regard to jurisdictional claims in published maps and institutional affiliations.

## References

[bib1] Silvio P Mariotti. Global Data on Visual Impairments. WHO: Geneva, 2012, pp. 1–17.

[bib2] Hejtmancik JF. Congenital cataracts and their molecular genetics. Semin Cell Dev Biol 2008; 19: 134–149. 18035564 10.1016/j.semcdb.2007.10.003 PMC2288487

[bib3] Shiels A, Bennett TM, Hejtmancik JF. Cat-Map: putting cataract on the map. Mol Vis 2010; 16: 2007–2015. 21042563 PMC2965572

[bib4] Shiels A, Hejtmancik JF. Mutations and mechanisms in congenital and age-related cataracts. Exp Eye Res 2017; 156: 95–102. 27334249 10.1016/j.exer.2016.06.011 PMC5538314

[bib5] Bu L, Jin Y, Shi Y, Chu R, Ban A. Mutant DNA-binding domain of HSF4 is associated with autosomal dominant lamellar and Marner cataract. Nat Genet 2002; 31: 276–278. 12089525 10.1038/ng921

[bib6] Morimoto RI. Cells in stress: transcriptional activation of heat shock genes. Science 1993; 259: 1409–1410. 8451637 10.1126/science.8451637

[bib7] Morimoto RI. Regulation of the heat shock transcriptional response: cross talk between a family of heat shock factors, molecular chaperones, and negative regulators. Genes Dev 1998; 12: 3788–3796. 9869631 10.1101/gad.12.24.3788

[bib8] Akerfelt M, Trouillet D, Mezger V, Sistonen L. Heat shock factors at a crossroad between stress and development. Ann NY Acad Sci 2007; 1113: 15–27. 17483205 10.1196/annals.1391.005

[bib9] Nakai A, Tanabe M, Kawazoe Y, Inazawa J, Morimoto RI, Nagata K. HSF4, a new member of the human heat shock factor family which lacks properties of a transcriptional activator. Mol Cell Biol 1997; 17: 469–481. 8972228 10.1128/mcb.17.1.469 PMC231772

[bib10] Tanabe M, Sasai N, Nagata K, Liu XD, Liu PC, Thiele DJ et al. The mammalian HSF4 gene generates both an activator and a repressor of heat shock genes by alternative splicing. J Biol Chem 1999; 274: 27845–27856. 10488131 10.1074/jbc.274.39.27845

[bib11] Somasundaram T, Bhat SP. Developmentally dictated expression of heat shock factors: exclusive expression of HSF4 in the postnatal lens and its specific interaction with alphaB-crystallin heat shock promoter. J Biol Chem 2004; 279: 44497–44503. 15308659 10.1074/jbc.M405813200

[bib12] Fujimoto M, Izu H, Seki K, Fukuda K, Nishida T. HSF4 is required for normal cell growth and differentiation during mouse lens development. EMBO J 2004; 23: 4297–4306. 15483628 10.1038/sj.emboj.7600435 PMC524399

[bib13] Min J, Zhang Y, Moskophidis D, Mivechi NF. Unique contribution of heat shock transcription factor 4 in ocular lens development and fiber cell differentiation. Genesis 2004; 40: 205–217. 15593327 10.1002/gene.20087

[bib14] Shi X, Cui B, Wang Z, Weng L, Xu Z. Removal of Hsf4 leads to cataract development in mice through down-regulation of γS-crystallin and Bfsp expression. BMC Mol Biol 2009; 10: 10. 19224648 10.1186/1471-2199-10-10 PMC2653017

[bib15] Zhou L, Zhang Z, Zheng Y, Zhu Y, Wei Z, Xu H et al. SKAP2, a novel target of HSF4b, associates with NCK2/F-actin at membrane ruffles and regulates actin reorganization in lens cell. J Cell Mol Med 2011; 15: 783–795. 20219016 10.1111/j.1582-4934.2010.01048.x PMC3922667

[bib16] Mou L, Xu JY, Li W, Lei X, Wu Y, Xu G et al. Identification of vimentin as a novel target of HSF4 in lens development and cataract by proteomic analysis. Invest Ophthalmol Vis Sci 2010; 51: 396–404. 19628735 10.1167/iovs.09-3772

[bib17] Jing Z, Gangalum RK, Bhat AM, Nagaoka Y, Jiang M, Bhat SP. HSF4 mutation p.Arg116His found in age-related cataracts and in normal populations produces childhood lamellar cataract in transgenic mice. Hum Mutat 2014; 35: 1068–1071. 24975927 10.1002/humu.22610 PMC4134754

[bib18] Gangalum RK, Jing Z, Bhat AM, Lee J, Nagaoka Y, Deng SX et al. Expression of the HSF4 DNA binding domain-EGFP hybrid gene recreates early childhood lamellar cataract in transgenic mice. Invest Ophthalmol Vis Sci 2014; 55: 7227–7240. 25168898 10.1167/iovs.14-14594 PMC4231995

[bib19] Yan Q, Liu J, Wan-Cheng LiD. Apoptosis in lens development and pathology. Differentiation 2006; 74: 195–211. 16759286 10.1111/j.1432-0436.2006.00068.x

[bib20] Dahm R. Lens fibre cell differentiation - a link with apoptosis? Ophthalmic Res 1999; 31: 163–183. 10224500 10.1159/000055530

[bib21] Pan H, Griep AE. Altered cell cycle regulation in the lens of HPV-16 E6 or E7 transgenic mice: implications for tumor suppressor gene function in development. Genes Dev 1994; 8: 1285–1299. 7926731 10.1101/gad.8.11.1285

[bib22] Nakamura T, Pichel JG, Williamssimons L, Westphal H. An apoptotic defect in lens differentiation caused by human p53 is rescued by a mutant allele. Proc Natl Acad Sci USA 1995; 92: 6142–6146. 7597093 10.1073/pnas.92.13.6142 PMC41658

[bib23] Pan H, Griep AE. Temporally distinct patterns of p53-dependent and p53-independent apoptosis during mouse lens development. Genes Dev 1995; 9: 2157–2169. 7657167 10.1101/gad.9.17.2157

[bib24] Geatrell JC, Mui Iryn Gan P, Mansergh FC, Kisiswa L, Jarrin M, Williams LA et al. Apoptosis gene profiling reveals spatio-temporal regulated expression of the p53/Mdm2 pathway during lens development. Exp Eye Res 2009; 88: 1137–1151. 19450442 10.1016/j.exer.2009.01.020 PMC2706329

[bib25] Deng M, Chen P, Liu F, Fu S, Tang H, Fu Y et al. The p53-Bak apoptotic signaling axis plays an essential role in regulating differentiation of the ocular lens. Curr Mol Med 2012; 12: 901–916. 22671997 10.2174/156652412802480899

[bib26] Liu FY, Tang XC, Deng M, Chen P, Ji W, Zhang X et al. The tumor suppressor p53 regulates c-Maf and Prox-1 to control lens differentiation. *Curr Mol Med* 2012; 12: 917–928. 22827438 10.2174/156652412802480835

[bib27] Wang WL, Li Q, Xu J, Cvekl A. Lens fiber cell differentiation and denucleation are disrupted through expression of the N-terminal nuclear receptor box of NCOA6 and result in p53-dependent and p53-independent apoptosis. Mol Biol Cell 2010; 21: 2453–2468. 20484573 10.1091/mbc.E09-12-1031 PMC2903674

[bib28] Babizhayev MA, Li DW, Kasus JA, Zoric L, Alió JL (eds). Roles of p53 in Ocular Development and Pathogenesis. Studies on the Cornea and Lens. Chapter 15, Springer Publisher, Inc,: NY, USA, p275–285..

[bib29] Fromm L, Overbeek PA. Inhibition of cell death by lens-specific overexpression of bcl-2 in transgenic mice. Dev Genet 1997; 20: 276–287. 9216067 10.1002/(SICI)1520-6408(1997)20:3<276::AID-DVG10>3.0.CO;2-6

[bib30] Wride MA, Parker E, Sanders EJ. Members of the bcl-2 and caspase families regulate nuclear degeneration during chick lens fibre differentiation. Dev Biol 1999; 213: 142–156. 10452852 10.1006/dbio.1999.9375

[bib31] Sanders EJ, Parker E. Retroviral overexpression of bcl-2 in the embryonic chick lens influences denucleation in differentiating lens fiber cells. Differentiation 2003; 71: 425–433. 12969335 10.1046/j.1432-0436.2003.7107005.x

[bib32] Mao Y, Liu J, Xiang H, Li DW. Human αA- and αB-crystallins bind to Bax and Bcl-XS to sequester their translocation during staurosporine-induced apoptosis. Cell Death Differ 2004; 11: 512–526. 14752512 10.1038/sj.cdd.4401384

[bib33] Weber GF, Menko AS. The canonical intrinsic mitochondrial death pathway has a non-apoptotic role in signaling lens cell differentiation. J Biol Chem 2005; 280: 22135–22145. 15826955 10.1074/jbc.M414270200

[bib34] Ishizaki Y, Jacobson MD, Raff MC. A role for caspases in lens fiber differentiation. J Cell Biol 1998; 140: 153–158. 9425163 10.1083/jcb.140.1.153 PMC2132591

[bib35] Li DW, Xiang H, Mao YW, Wang J. Caspase-3 is a major caspase mediating apoptosis during rat lens development and cataract formation. Invest Ophthalmol Vis Sci 41: S323.

[bib36] Li DW, Xiang H, Mao Y, Wang J, Fass U, Zhang X et al. Caspase-3 is actively involved in okadaic acid-induced lens epithelial cell apoptosis. Exp Cell Res 2001; 266: 279–291. 11399056 10.1006/excr.2001.5223

[bib37] Lee A, Morrow JS, Fowler VM. Caspase remodeling of the spectrin membrane skeleton during lens development and aging. J Biol Chem 2001; 276: 20735–20742. 11278555 10.1074/jbc.M009723200

[bib38] Foley JD, Rosenbaum H, Griep AE. Temporal regulation of VEID-7-amino-4-trifluoromethylcoumarin cleavage activity and caspase-6 correlates with organelle loss during lens development. J Biol Chem 2004; 279: 32142–32150. 15161922 10.1074/jbc.M313683200

[bib39] Morozov V. Caspase-dependent secondary lens fiber cell disintegration in A-/ B-crystallin double-knockout mice. Development 2006; 133: 813–821. 16439475 10.1242/dev.02262

[bib40] Yamashita M, Mizusawa N, Hojo M, Yabu T. Extensive apoptosis and abnormal morphogenesis in pro-caspase-3 transgenic zebrafish during development. J Exp Biol 2008; 211: 1874–1881. 18515717 10.1242/jeb.012690

[bib41] Liu J, Schlosser R, Ma W, Dong Z, Feng H, Liu L et al. Human αA- and αB-crystallins prevent UVA-induced apoptosis through regulation of PKCα, RAF/MEK/ERK and AKT signaling pathways. Exp Eye Res 2004; 79: 393–403. 10.1016/j.exer.2004.06.015 15336502

[bib42] Mehlen P, Kretz-Remy C, Preville X, Arrigo AP. Human hsp27, Drosophila hsp27 and human alphaB-crystallin expression-mediated increase in glutathione is essential for the protective activity of these proteins against TNFalpha-induced cell death. EMBO J 1996; 15: 2695–2706. 8654367 PMC450205

[bib43] Mehlen P, Schulze-Osthoff K, Arrigo AP. Small stress proteins as novel regulators of apoptosis. Heat shock protein 27 blocks Fas/APO-1- and staurosporine-induced cell death. J Biol Chem 1996; 271: 16510. 8663291 10.1074/jbc.271.28.16510

[bib44] Andley UP, Song Z, Wawrousek EF, Fleming TP, Bassnett S. Differential protective activity of αA- and αB-crystallin in lens epithelial cells. J Biol Chem 2000; 275: 36823–36831. 10967101 10.1074/jbc.M004233200

[bib45] Li DW, Liu JP, Mao YW, Xiang H, Wang J, Ma WY et al. Calcium-activated RAF/MEK/ERK signaling pathway mediates p53-dependent apoptosis and is abrogated by alpha B-crystallin through inhibition of RAS activation. Mol Biol Cell 2005; 16: 4437–4453. 16000378 10.1091/mbc.E05-01-0010 PMC1196350

[bib46] Wride MA, Sanders EJ. Nuclear degeneration in the developing lens and its regulation by TNFα. Exp Eye Res 1998; 66: 371–383. 9533864 10.1006/exer.1997.0440

[bib47] Wiley LA, Rajagopal R, Dattilo LK, Beebe DC. The tumor suppressor gene Trp53 protects the mouse lens against posterior subcapsular cataracts and the BMP receptor Acvr1 acts as a tumor suppressor in the lens. Dis Model Mech 2011; 4: 484–495. 21504908 10.1242/dmm.006593 PMC3124053

[bib48] Reichel MB, Ali RR, D'Esposito F, Clarke AR, Luthert PJ, Bhattacharya SS et al. High frequency of persistent hyperplastic primary vitreous and cataracts in p53-deficient mice. Cell Death Differ 1998; 5: 156–162. 10200460 10.1038/sj.cdd.4400326

[bib49] Ji WK, Tang XC, Yi M, Chen PQ, Liu FY, Hu XH et al. p53 directly regulates alphaA- and betaA3/A1-crystallin genes to modulate lens differentiation. Curr Mol Med 2013; 13: 968–978. 23745585 10.2174/15665240113139990052

[bib50] Hu XH, Nie Q, Yi M, Li TT, Wang ZF, Huang ZX et al. The tumor suppressor, p53 regulates the gammaA-crystallin gene during mouse lens development. Curr Mol Med 2014; 14: 1197–1204. 25336329 10.2174/1566524014666141021144927

[bib51] Zandy AJ. Role of the executioner caspases during lens development. J Biol Chem 2005; 280: 30263–30272. 15994297 10.1074/jbc.M504007200

[bib52] Huang M, Li D, Huang Y, Cui X, Liao S, Wang J et al. HSF4 promotes G1/S arrest in human lens epithelial cells by stabilizing p53. Biochim Biophys Acta 2015; 1853: 1808–1817. 25940838 10.1016/j.bbamcr.2015.04.018

[bib53] Doyle EL, Booher NJ, Standage DS, Voytas DF, Brendel VP, VanDyk JK et al. TAL Effector-Nucleotide Targeter (TALE-NT) 2.0: tools for TAL effector design and target prediction. Nucleic Acids Res 2012; 40: W117–W122. 22693217 10.1093/nar/gks608 PMC3394250

[bib54] Swan CL, Evans TG, Sylvain N, Krone PH. Zebrafish HSF4: a novel protein that shares features of both HSF1 and HSF4 of mammals. Cell Stress Chaperones 2012; 17: 623–637. 22528049 10.1007/s12192-012-0337-3 PMC3535164

[bib55] Dahm R, Gribbon C, Quinlan RA, Prescott AR. Changes in the nucleolar and coiled body compartments precede lamina and chromatin reorganization during fibre cell denucleation in the bovine lens. Eur J Cell Biol 1998; 75: 237–246. 9587055 10.1016/S0171-9335(98)80118-0

[bib56] Sanwal M, Muel AS, Chaudun E, Courtois Y, Counis MF. Chromatin condensation and terminal differentiation process in embryonic chicken lens *in vivo* and *in vitro*. Exp Cell Res 1986; 167: 429–439. 3770096 10.1016/0014-4827(86)90183-7

[bib57] Cui X, Liu H, Li J, Guo K, Han W, Dong Y et al. Heat shock factor 4 regulates lens epithelial cell homeostasis by working with lysosome and anti-apoptosis pathways. Int J Biochem Cell Biol 2016; 79: 118–127. 27586257 10.1016/j.biocel.2016.08.022

[bib58] Watanabe G, Kato S, Nakata H, Ishida T, Ohuchi N, Ishioka C. αB-crystallin: a novel p53-target gene required for p53-dependent apoptosis. Cancer Sci 2009; 100: 2368–2375. 19799611 10.1111/j.1349-7006.2009.01316.x PMC11159724

[bib59] Yang X, Wang J, Liu C, Grizzle WE, Yu S, Zhang S et al. Cleavage of p53-vimentin complex enhances tumor necrosis factor-related apoptosis-inducing ligand-mediated apoptosis of rheumatoid arthritis synovial fibroblasts. Am J Pathol 2005; 167: 705–719. 16127151 10.1016/S0002-9440(10)62045-7 PMC1698724

[bib60] Hueber A, Eichholtz CD, Kociok N, Esser JM, Esser PJ. Lens epithelial cells express CD95 and CD95 ligand treatment induces cell death and DNA fragmentation *in vitro*. Eur J Ophthalmol 2003; 13: 241–245. 12747644 10.1177/112067210301300301

[bib61] Zhao B, Mei Y, Schipma MJ, Roth EW, Bleher R, Rappoport JZ et al. Nuclear condensation during mouse erythropoiesis requires caspase-3-mediated nuclear opening. Dev Cell 2016; 36: 498–510. 26954545 10.1016/j.devcel.2016.02.001 PMC4785602

[bib62] Hu WF, Gong L, Cao Z, Ma H, Ji W, Deng M et al. AlphaA- and alphaB-crystallins interact with caspase-3 and Bax to guard mouse lens development. Curr Mol Med 2012; 12: 177–187. 22280356 10.2174/156652412798889036

