# Supplementary material for: HSF4 regulates lens fiber cell differentiation by activating p53 and its downstream regulators
Source: Cell Death Dis. 2017 Oct 5;8(10):e3082–. doi: 10.1038/cddis.2017.478 (PMC5682647; doi:10.1038/cddis.2017.478)
Supplement: Supplementary Information [file cddis2017478x1.docx]

## Supplemental Information:

S1. Zebrafish hsf4 was expressed in eyes after 48 hpf.


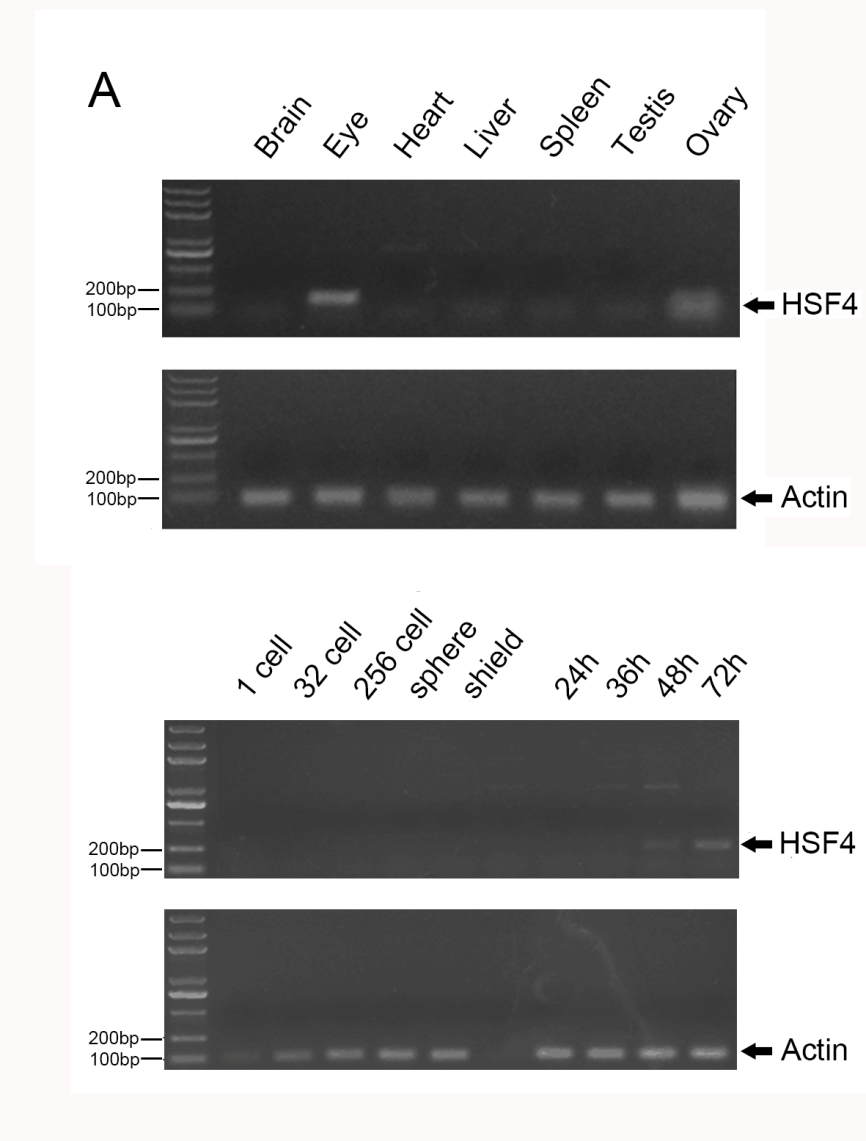


Figure S1. Spatial and temporal expression of HSF4 was detected by Semi-quantitative PCR. The house keeping gene actin used as a internal reference. HSF4 was highly expressed at eyes after 48hpf.

S2 and S3. The lens epithelium cells were overproliferated in hsf4^null^ zebrafish.


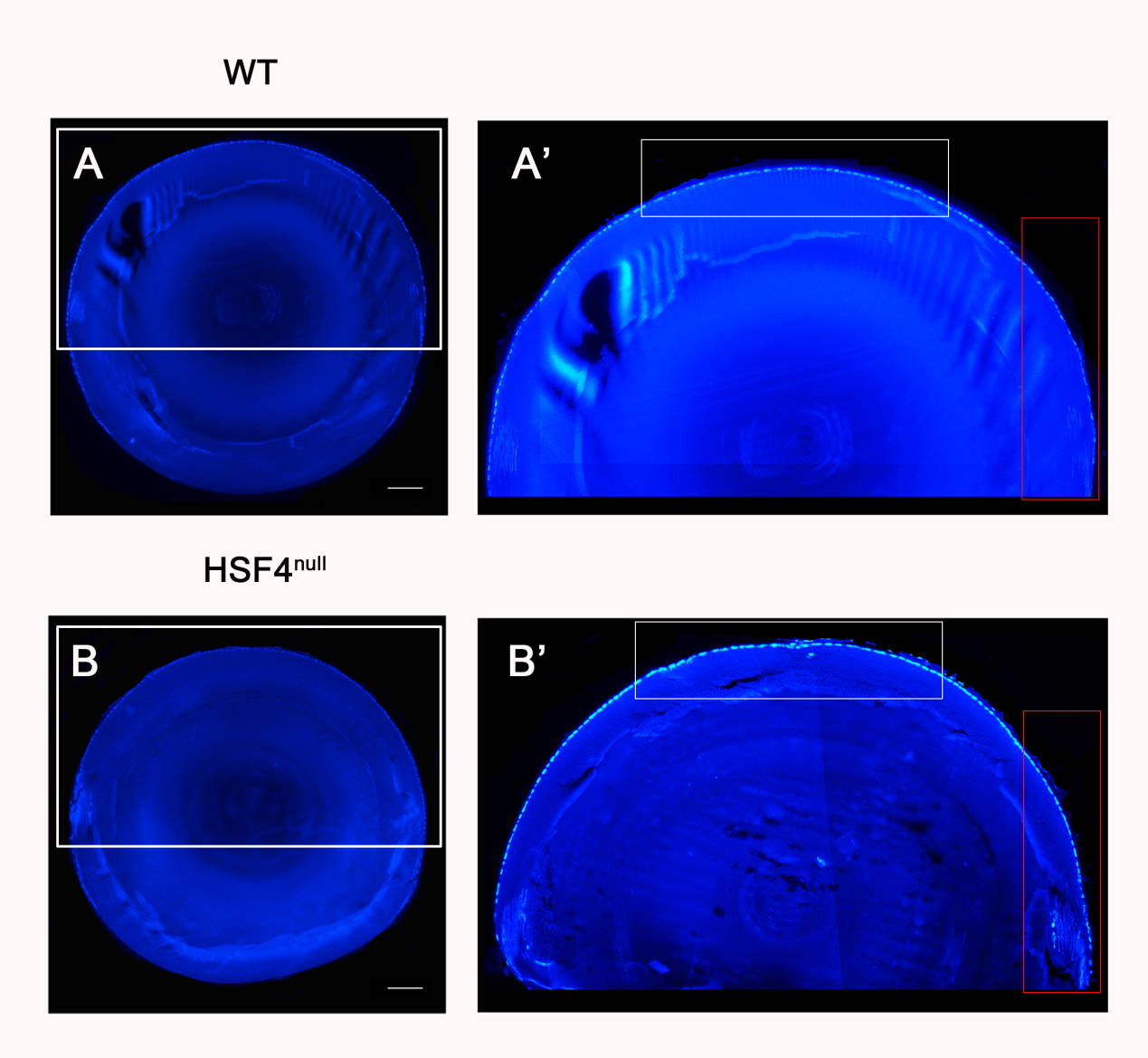


Figure S2. Overproliferation of lens epithelium cells caused by knockout of HSF4 in zebrafish lens. DAPI staining the paraffin section of 5M old WT (A) and hsf4^null^ (B) zebrafish lens. The depth of each paraffin section was 8um. The enlarge images were listed at the right line. The DAPI staining result showed that there are more epithelial cells existed in the hsf4^null^ zebrafish lens (B’) rather than the WT lens (A’). Scale bar 500μm.


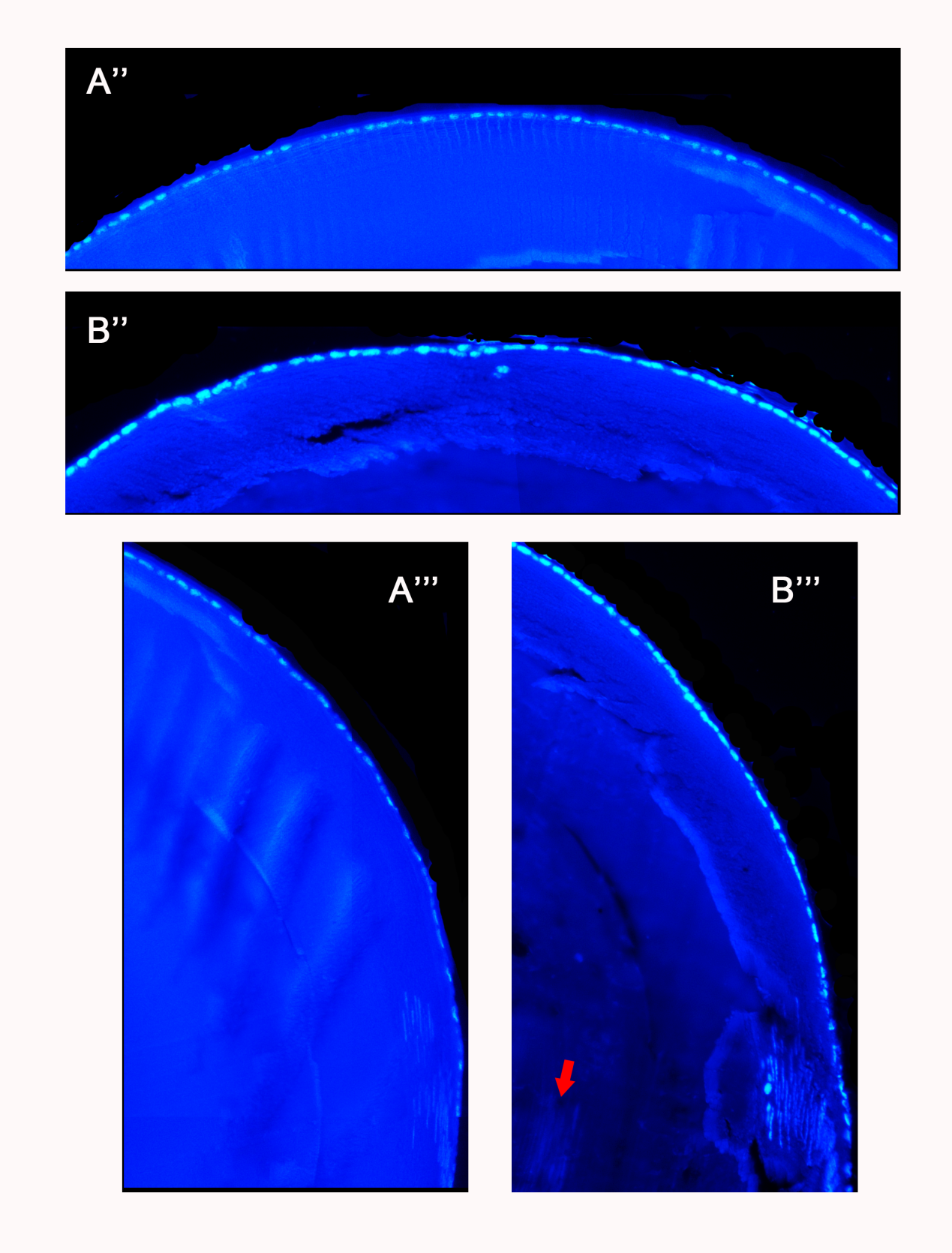


Figure S3. Enlarged images of the box in figure S2 A’ ans B’. The white box indicated the anterior region and the red box indicated the germinative zone of the lens. By comparing the nucleuses, we found out that there are more epithelial cells existed in the hsf4^null^ zebrafish lens (B’’) and (B’’’) than in the WT lens (A’’) and (A’’’). The red arrow indicated the nucleuses deposited in the hsf4^null^ zebrafish lens.

S4. The arrangement of the lens fiber cells can be visualized by phalloidin immunofluorescence
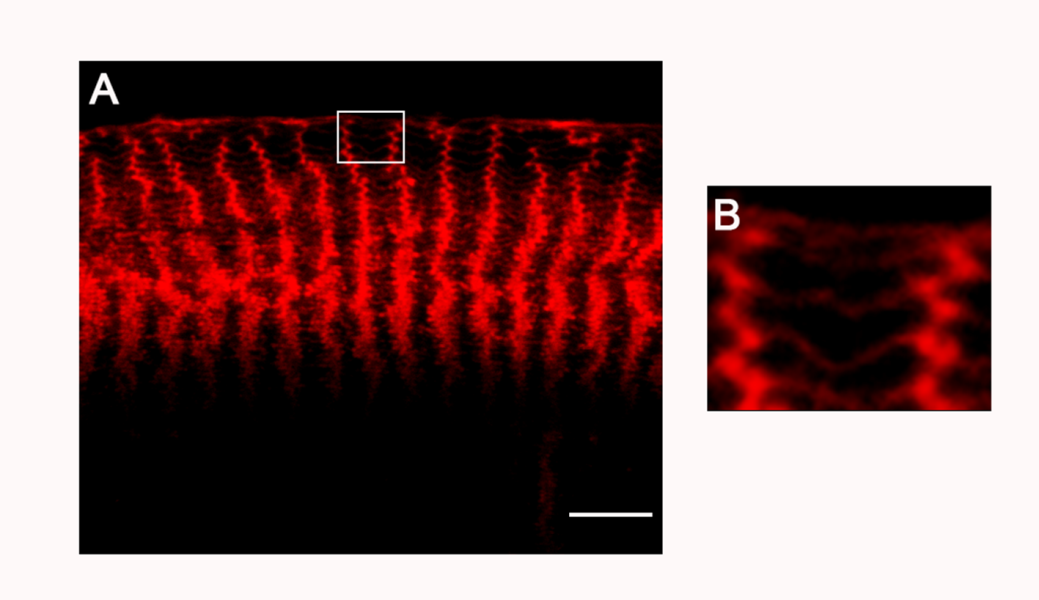


Figure S4. Immunofluorescence staining of the lens fiber cells using phalloidin antibodies. (A)The mature lens fiber cell is hexagonal in shape. Along its membrane arranged the actin bundles. When cross sectioned, we can find the actin bundles are arranged in three distinct units, one in each fiber cell. These structures can be labeled by phalloidin immunofluorescence staining. Through microscopy observation, we observed each fiber cell can be label as ahexagonal fluorescence circle (B) and these circles stack together forming a regular structure. (B) Enlargement picture of the with box in picture A. Scale bar: 10μm.

S5. Validation HSF4 overexpression and interfering efficiency through real-time PCR detection.


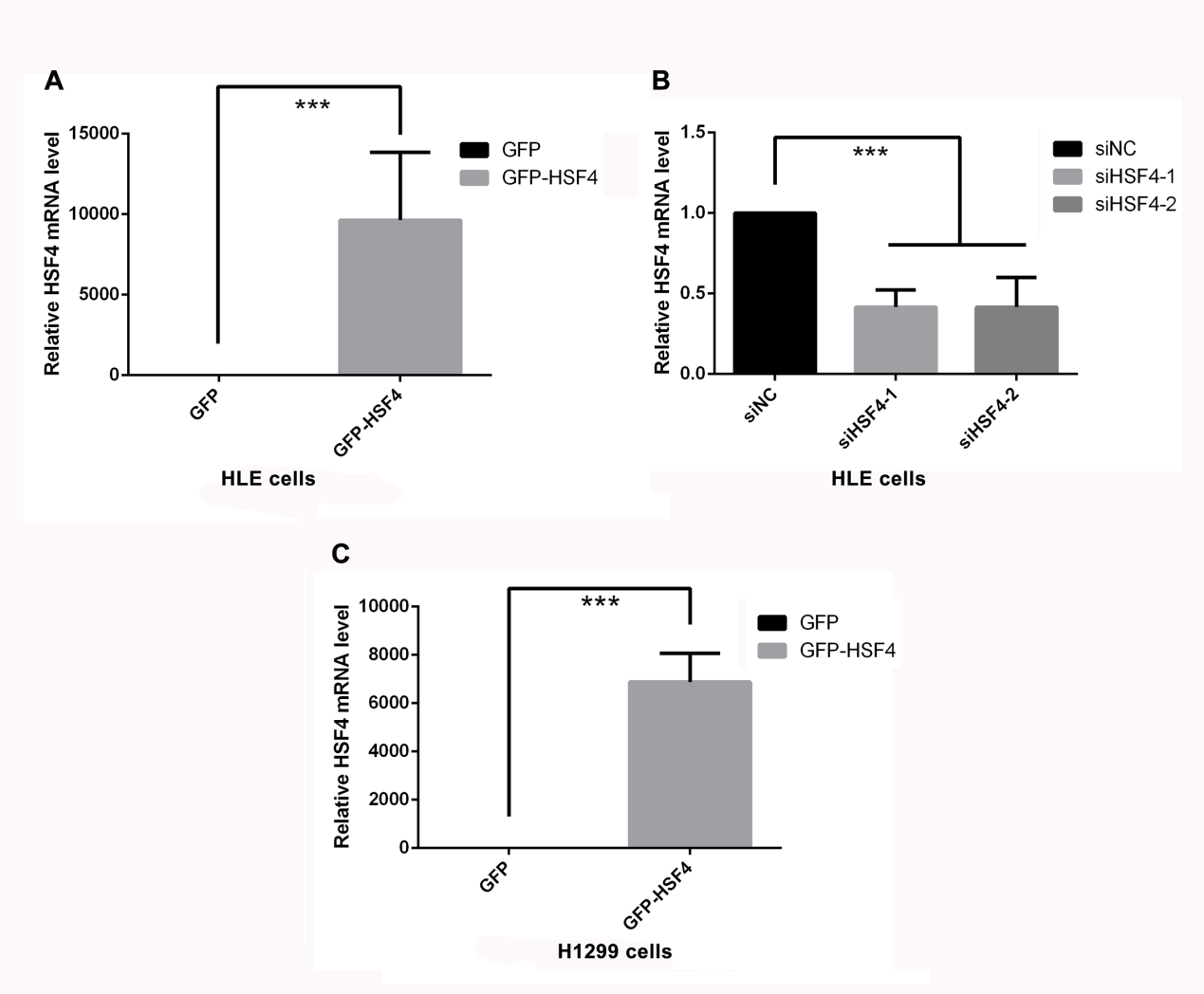


Figure S5. Verification of HSF4 overexpression and interfering effect at RNA level. (A) and (C) GFP-HSF4 plasmids and the negative control GFP plasmids were transfected into HLE cells(A) and H1299 cells(C) respectively. After 48h, cells were harvested for RNA extraction. The HSF4 mRNA significantly increased in the cells transfected with GFP-HSF4 plasmids through the reverse-transcription and real-time PCR detection. (B) HLE cells were transfected with two pairs of HSF4 specific siRNA and the negative control siNC. The cells were harvested for RNA extraction 48h after the transfection. Both pairs of the HSF4 specific siRNA down-regulate the HSF4 mRNA to about 50% through the reverse-transcription and real-time PCR detection. For the real-time PCR detection, the relative mRNA levels were normalized to β-actin. Three independent experiments were performed. ***, p < 0.001.

S6. Validation HSF4 overexpression and interfering efficiency through Western-blot detection.


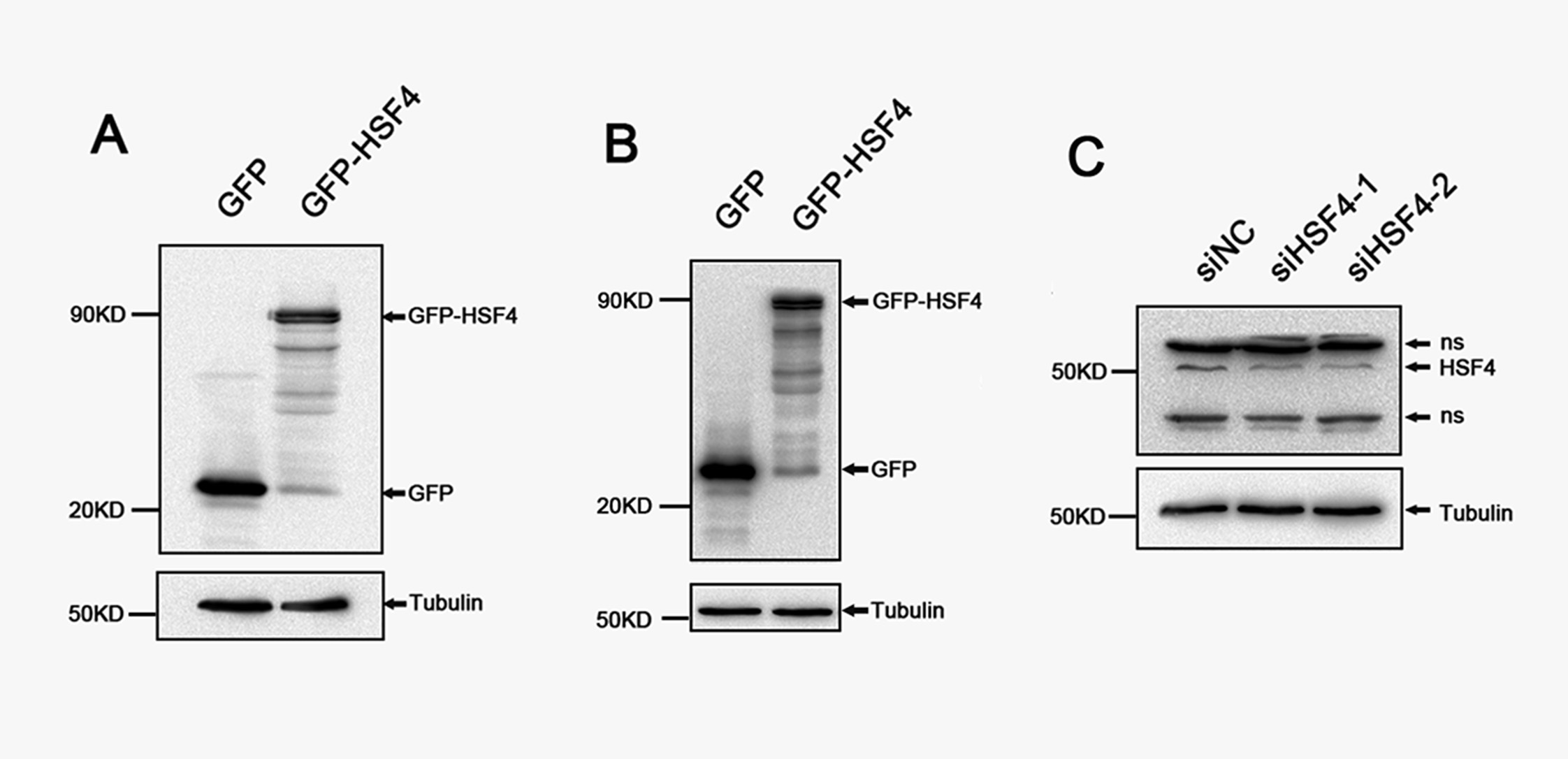


Figure S6. Verification of HSF4 overexpression and interfering effect at protein level. (A) and (B) Western bolt detection indicated that GPF-HSF4 existed in the HLE cells (A) and H1299 cells (B) transfected with GFP-HSF4 plasmids. (C) Western bolt detection indicated that the HSF4 specific siRNA siHSF4-1 and siHSF4-2 can decrease the HSF4 in the HLE cells.

S7. Detection of the αB-crystallin expression level in the hsf4^null^ zebrafish


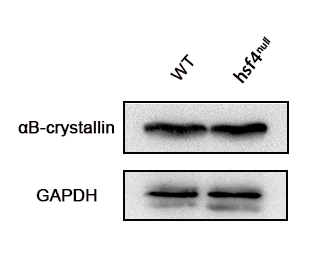


Figure S7. Western blot examination of the expression level of αB-crystallin in hsf4^null^ zebrafish. 6M old WT and hsf4^null^ zebrafish lenses were isolated and subjected to protein extraction. The result from western blot detection indicated that the expression of αB-crystallin was constant in the hsf4^null^ zebrafish.
